# Flexural strength and surface hardness in titanium nanoparticle-reinforced polymethyl methacrylate provisional restorative material: An *in vitro* study

**DOI:** 10.6026/973206300222572

**Published:** 2026-04-30

**Authors:** Nikhil K, Ravi Kumar C, Chalapathi Rao D, Ravi Shankar Y, Harilal G

**Affiliations:** 1 Department of Prosthodontics, Mamata Dental College, Khammam, Telangana, India

**Keywords:** Provisional polymethyl methacrylate resin (PPMMAR), titanium nanoparticles, provisional restoration, flexural strength, surface hardness

## Abstract

Polymethyl methacrylate (PMMA) provisional materials often suffer from inadequate mechanical properties, particularly low flexural strength and surface hardness, limiting their durability. Therefore, it is of interest to investigate whether adding 3 wt% titanium dioxide (TiO_2_) nanoparticles could improve these properties in heat-cured PMMA resin. Hence, a total of 160 standardized specimens were tested for flexural strength and Vickers hardness using universal testing and micro-hardness methods. TiO_2_-reinforced PMMA showed a significant increase in flexural strength (56.62 ± 1.43 to 70.65 ± 8.32 MPa) and surface hardness (23.22 ± 1.30 to 26.74 ± 1.03 VH). Incorporating 3 wt% TiO_2_ notably enhances PMMA's mechanical performance, supporting its use in more demanding provisional applications.

## Background:

Loss of teeth is it due to trauma or disease, has been a thorny issue in the field of dentistry and has remained a source of innovation in the field of prosthetic materials [1]. The search of the best artificial replacements of the lost teeth continues to be one of the main concerns of studies on restorative and prosthetic dentistry. The replacement of teeth is done either by using removable or fixed dental prostheses to ensure that patients have functional and aesthetic harmony and fixed ones tend to be preferred by patients due to their convenience [2]. Temporary or provisional reconstructions are common between the process of tooth preparation and polishing of the permanent prosthesis [3]. These provisional restorations play a crucial purpose in the diagnosis of aesthetic and functional results. Perfective provisional restorations safeguard the pulp, uphold the health of the gingiva, stop the development of plaque and gingival hyperplasia, support oral hygiene, direct regeneration of supportive prosthetic tissues and provide stability of the adjacent and opposing dentition [4]. They are also useful in determining the fantasised role and shape of the final prosthesis [5]. The provisional restorations should endure functional chewing loads especially complex clinical scenarios like full mouth rehabilitation with reduced vertical dimension, long-span bridges, temporomandibular joint appliances, parafunctional habits and implant-supported restorations that may be subjected to prolonged use [6]. Mechanical durability on such situations becomes a key to clinical success. Today, the most popular non-metallic substance used in the field of prosthodontics is provisional polymethyl methacrylate resin (PPMMAR) that was initially developed by Dr. Walter Wright in 1937 [7]. PPMMAR is used as it is easy to manipulate, cheap, biocompatible, aesthetics, dimensional stable, marginally well adapted and can be polished [8]. In spite of such benefits, PPMMAR possesses some weaknesses namely, residual monomer sensitivity, low mechanical strength, fracture sensitivity, low hardness, colour stability, porosity and deformation [9]. Of particular significance are surface hardness and flexural strength because decreased values lead to microbial adhesion and material failure [10]. In order to overcome these shortcomings, various experimental methods have tried to improve the physical and mechanical characteristics of PPMMAR.

The use of metal oxides including zirconium, silver, copper and titanium dioxide has been one of the promising directions [11]. More recently nanotechnology has taken a centre stage and nanoparticles have been incorporated into polymer matrices in large quantities to improve strength and surface characteristics considerably [12]. Nanotechnology is one of the significant scientific developments of the twenty-first century which entails manipulation of matter at atomic or molecular levels [13]. It has transformed the manner in which material sciences have been done by enhancing the mechanical, physical and biological performance of dental materials. The main distinction between nanoparticles and traditional fillers is their size and morphology and they can be spherical, tubular, rod-shaped or irregular and they can be single or agglomerated clusters [14]. Titanium dioxide nanoparticles (TiO_2_ NPs) have very good mechanical characteristics such as a high modulus of elasticity of about 230 Gpa [15]. They have been found to improve the structural integrity and antimicrobial effect on their incorporation into dental materials [16]. Nevertheless, problems like agglomeration have to be overcome, which can be solved in most cases by applying surface coating like silanization in order to have a homogeneous dispersion. TiO_2_ nano-particles are biocompatible, white, chemically stable and resistant to corrosion, non-toxic and cheap [17]. They can be used to enhance the electrical, optical, chemical and mechanical behaviour of a material. Although titanium dioxide nanoparticles have a promising future, there has been minimal research on how to integrate them into PPMMAR [18]. There is an urgent necessity to assess their effects on such major mechanical properties as flexural strength and surface hardness. This type of studies might help in the formation of more robust and sturdier provisional materials that can be used over a long period in clinical settings [19]. Therefore, it is of interest to describe advances in provisional restorative substances to provide improved functionality and durability in various prosthodontic scenarios.

## Materials and Methods:

This was an *in vitro* study carried out in the Department of Prosthodontics, Mamata Dental College, Khammam, Telangana, India. It was approved by the Institutional Ethics Committee.

## Grouping of samples:

A total of 160 samples were fabricated and divided into two main groups. Each main group consisted of 80 samples, further subdivided into two subgroups, each containing 40 samples.

## Study procedure:

The materials used were heat-polymerized provisional acrylic resin, titanium dioxide nanoparticles and dental stone. Three brass metals dies of dimensions 65 mm in length, 10 mm in width and 3 mm in height were fabricated according to ISO 1567-1999 standards. Gypsum was moulded with uniform mould gaps and sample replica blocks were fabricated.

## Fabrication of specimens:

For the control group (Group I), 40 specimens of conventional heat-cured PPMMAR were fabricated using metal analogues (65 x 10 x 3 mm) invested in dental stone within brass flasks. After applying separating media, the moulds were prepared with vacuum-mixed dental stone. PPMMAR was mixed at a ratio of 5 g powder to 3.5 ml monomer, packed at dough stage and pressed at 1500 PSI. Polymerization was carried out at 74°C for 2 hours followed by 100°C for 1 hour. The specimens were deflasked, finished with tungsten carbide burs and sandpaper and stored in distilled water. For the test group (Group II), 80 specimens were fabricated by incorporating 3 wt% titanium dioxide nanoparticles (3 g per 100 g of polymer) into PPMMAR. Nanoparticles were first mixed with the polymer using a mortar and pestle. The modified powder was then processed identically to the control group. Specimens were finished and polished as before, ensuring standard dimensions and preserved in distilled water.

## Testing for flexural strength:

Flexural strength testing was performed using an Instron Universal Testing Machine. A total of 80 samples, 40 from each group, were tested in a load cell with a 250 kN capacity. A vertical force was applied at the specimen's midpoint by a chisel at a crosshead speed of 5 mm/min and a span length of 40 mm until specimen fracture occurred under flexural loading. Fracture was indicated by a sharp crackling sound accompanied by a sudden drop in the digital signal. The load required to induce failure was recorded in MPa. Results were recorded using a three-point bending test.

The flexural strength was calculated using the equation:

S = 3pl / 2bd^2^

Where S is the flexural strength, p is the force at the fracture point, l is the distance between the two supports, b is the specimen's width and d is the specimen's thickness.

## Testing for hardness:

The Vickers method was utilized to assess surface hardness according to the Vickers microhardness testing procedure (ASTM E-384). A diamond indenter with a square-based pyramidal shape was employed, applying light loads to produce an indentation whose dimensions were optically measured and converted into a hardness value. Test specimens were meticulously polished to clearly define the indentation and positioned perpendicular to the indenter during testing. Specimens were mounted in a plastic medium to aid preparation and handling.

## Statistical analysis:

Statistical analyses were performed using the Statistical Package for Social Sciences (SPSS) for Windows, version 26.0. Descriptive analysis included the expression of flexural strength and surface hardness in terms of mean and standard deviation. Mean and standard deviation scores were compared across study groups using the one-way ANOVA test. Multiple comparisons within study groups were conducted using the independent t-test. Statistical significance was set at p < 0.05.

## Results:

The maximum flexural strength mean value of 70.65 MPa was observed in Group II (PPMMAR + 3 wt% TiO_2_), while the minimum mean value of 56.62 MPa was observed in Group I (unmodified PPMMAR) (Tables 1 and 2 - see PDF). The flexural strength of PMMA significantly improved with the incorporation of titanium dioxide nanoparticles. This difference was statistically significant (p = 0.0001), as revealed by an independent t-test (t = 10.5113) (Table 3 - see PDF). The maximum surface hardness mean value of 26.74 VH was observed in Group II, while the minimum mean value of 23.22 VH was observed in Group I (Table 4 - see PDF). The independent t-test revealed this difference to be statistically significant (t = -13.4366, p = 0.0001) (Table 5 - see PDF).

## Discussion:

The reason as to why polymethyl methacrylate is still extensively utilized in provisional restorations is because of their affordability, manipulability, lightness, acceptable aesthetics and oral environment stability [1, 2]. But temporary PMMA undergoes a multidirectional force when mastication takes place and it should have enough mechanical properties especially flexural strength and surface hardness to withstand fracture and wear in clinical services [3]. Provisional prostheses have a vital characteristic known as flexural strength, particularly in long span bridges or full-mouth rehabilitations, masticatory stress at all times may cause microcracks in materials which eventually result in material failure [4]. Hardness on the surface indicates resistance to abrasion and wear, maintenance of marginal integrity and minimization of plaque deposits and discoloration [5]. The surface hardness of most provisional resins is intrinsically low and this may put the prosthesis at risk in the long run. In order to guarantee long-term clinical performance, improvement of both these properties is thus necessary [6]. Several methods have been investigated to enhance the mechanical characteristics of PMMA such as copolymerization and addition of metallic oxides [7]. The use of titanium dioxide nanoparticles has been the most investigated of these methods because of its desirable properties such as high chemical stability, antimicrobial property, low toxicity, cost-effectiveness and bonding tendency with the resin matrix [8]. Nanotechnology also has some benefits in that it presents nano-size fillers that have the potential to enhance the mechanical, optical and antimicrobial properties of restorative materials [9]. Due to the high surface area to volume ratio of nanoparticles, they bond well and this leads to the polymer matrix being better reinforced [10]. TiO_2_ nanoparticles, in the form of spheres, are especially useful in the process of improving the strength and surface properties of dental polymers. The use of heat-cure PMMA in this research compared to cold-cure varieties was because the former had better mechanical characteristics and it contained lesser amounts of monomers [11]. The test samples were prepared based on the ISO 1567-1999 guidelines [12]. The control group of unmodified PMMA and test group of PMMA modified with 3 wt% TiO_2_ nanoparticles all underwent a standardized short cycle polymerization process. The TiO_2_ nanoparticles were safely incorporated in the powder of the polymer before it was mixed with the monomer. Each mechanical property was tested on 80 samples, 40 samples per group. Flexural strength was measured by using a Universal Testing Machine and surface hardness was measured using a Micro Vickers hardness tester. The rigorous statistical analysis was done on all results. The average flexural strength of the control was 56.62 with standard deviation at 1.43 Mpa and the TiO_2_ reinforced was 70.65 with standard deviation value 8.32 Mpa. The difference between them was statistically significant (p < 0.001) and exceeded the minimum clinical requirement of 50 MPa set by international standards [13]. Previous studies have shown such improvements with equivalent concentrations of TiO_2_ and those with 2.5 wt% TiO_2_ had significant changes in flexural properties [14]. The process behind this enhancement is credited to transformation toughening by which TiO_2_ switches to tetragonal to monoclinic phase transition which captures energy and halts crack propagation over the polymer matrix [15]. It has also been found by research which investigated higher levels of TiO_2_ at 5 wt percent that flexural strength was enhanced and the enhancement was due to the chemical interactions between TiO_2_ and the ester bonds within PMMA [16]. It is possible that the nanoparticles inhibit chain mobility and are cross-linking agents, which strengthen the matrix structure. It has though been continuously noted that overloading of fillers which surpasses 5 wt can also result in low mechanical performance when there is poor dispersion and agglomeration of particles in the resin [17]. Experiments involving concentration-dependent effects have shown gradual enhancement in flexural strength with the addition of TiO_2_ nanoparticles to a maximum level [18]. These improvements are probably attributed to the improvements in the interfacial shear strength and the cross-linking of resin and nanofillers. Nanoparticles should be uniformly dispersed and should fully be wet by the resin to facilitate stress transfer and eliminate crack formation behavior at the filler-matrix interface [19]. On the surface hardness, the control group had the mean of 23.22 VH and the test group recorded a much higher value of 26.74 VH, which was statistically significant (p < 0.001). This increase is credited to homogenous scattering of nanoparticles between polymer chains and this limits segmental movement and increases abrasion resistance [20]. It has also been found out that even low levels of TiO_2_ nanoparticles 0.5 wt% and 1 wt% enhance hardness because it is able to penetrate the polymer network and lower molecular mobility. Several supramolecular bonds ensure high interfacial shear strength that ensures an increased resistance to abrasion [21]. Experiments that have studied the microhardness at 1 wt% percentage and 5 wt% percentage TiO_2_ found higher values and therefore indicated that the higher the microhardness, the higher the wear resistance [22]. It has however been observed that at high concentrations can decrease in impact strength because of poor distribution into the resin and colour stability can be compromised at TiO_2_ concentration above 5%. Other reports have indicated detrimental impacts on mechanical characteristics of nanoparticles at high concentrations which may be attributed to light scattering, decrease in cure depth, radical quenching, particle agglomeration and excess monomer content [21]. These results provide evidence of the need to optimize the concentration of nanoparticles when developing a balance between mechanical reinforcement and acceptable aesthetics and processing properties. The tribological behaviour of PMMA with TiO_2_ at different concentrations of 1, 3, 5 and 7 wt% has been studied, showing the best results with the area of 3 wt%, which has high tensile strength, wear resistance and smoothness of surfaces [23]. This has been enhanced by the fact that TiO_2_ inhibits crack propagation and fills surface irregularities as well as providing a denser polymer matrix. This result confirms the concentration that was chosen in the current work and also justifies the reason why 3 wt% was chosen as the best concentration to make use of as reinforcement. Addition of 3 wt% Titanium dioxide nanoparticles in PMMA leads to great enhancement in flexural strength as well as surface hardness. This is the best concentration to have a balance between mechanical enhancement and no negative effects on handling or aesthetic properties. This *in vitro* study outcome validates the possibility of using TiO_2_ reinforced PMMA in the provision of more durable and wear resistant provisional restoration. Although the study has had several drawbacks, such as simplified specimen geometry, limited property assessment, no analysis of chemical interactions, no clinical simulation and use of only spherical TiO_2_ nanoparticles with no surface modulations, the study showed a progressive increase in flexural strength and surface hardness of PPMMAR with addition of titanium dioxide nanoparticles. These findings hope to have a good future in enhancing mechanical performance in future clinical procedures. The reinforced provisionals have slightly greyish appearance, thus compromising their aesthetics, thus making them more appropriate to use in the teeth located in the rear. Conventional provisional PMMA resin reinforced with zirconia and titanium oxide nanoparticles exhibited significantly enhanced mechanical properties compared with newly marketed provisional PMMA materials [24].

## Conclusion:

We show that addition of 3 wt% titanium dioxide nanoparticles had a considerable positive effect on both flexural strength and surface hardness of the modified polymethyl methacrylate resin as compared to the standard one. These enhancements indicate that titanium nanoparticle reinforcement enhances the mechanical properties of PPMMAR positively and hence it has greater durability and could be used as a plausible long-term provisional restorative material in the field of prosthodontics.

## References

[1] Hamdy TM (2024). BMC Oral Health..

[2] Alhotan A (2023). Polymers (Basel)..

[3] Hamdy TM (2023). BMC Oral Health..

[4] El-Hussein IG (2024). J Contemp Dent Pract..

[5] Viswanathan AK, Krishnan R (2025). Clin Exp Dent Res..

[6] Anitha KV, Krishnan R (2024). J Indian Prosthodont Soc..

[7] Pai E (2023). J Indian Prosthodont Soc..

[8] Abdelraouf RM (2022). Polymers (Basel)..

[9] Rahaman Ali AAA (2020). J Prosthodont..

[10] Sasany R (2025). J Prosthet Dent..

[11] Li GH (2025). Clin Exp Dent Res..

[12] Bacali C (2020). Clin Oral Investig..

[13] Deb S (2020). J Contemp Dent Pract..

[14] Chhabra M (2022). J Oral Biol Craniofac Res..

[15] Kiran A (2020). J Ayub Med Coll Abbottabad..

[16] AlQahtani M, Haralur SB (2020). Medicina (Kaunas)..

[17] Kaur H (2022). J Contemp Dent Pract..

[18] Gonçalves NI (2020). J Mech Behav Biomed Mater..

[19] Raj V (2021). J Contemp Dent Pract..

[20] Al-Dwairi ZN (2023). J Prosthodont..

[21] Li GH (2021). J Mech Behav Biomed Mater..

[22] Bollepalli A (2025). J Indian Prosthodont Soc..

[23] Hamdy TM (2024). BMC Oral Health..

[24] Jehan A (2022). J Med Pharm Allied Sci..

